# Genetic Variation and Population Substructure in Outbred CD-1 Mice: Implications for Genome-Wide Association Studies

**DOI:** 10.1371/journal.pone.0004729

**Published:** 2009-03-06

**Authors:** Kimberly A. Aldinger, Greta Sokoloff, David M. Rosenberg, Abraham A. Palmer, Kathleen J. Millen

**Affiliations:** 1 Committee on Neurobiology, The University of Chicago, Chicago, Illinois, United States of America; 2 Department of Human Genetics, The University of Chicago, Chicago, Illinois, United States of America; 3 Department of Psychiatry and Behavioral Neuroscience, The University of Chicago, Chicago, Illinois, United States of America; 4 Department of Neurology, The University of Chicago, Chicago, Illinois, United States of America; Centre National de la Recherche Scientifique, France

## Abstract

Outbred laboratory mouse populations are widely used in biomedical research. Since little is known about the degree of genetic variation present in these populations, they are not widely used for genetic studies. Commercially available outbred CD-1 mice are drawn from an extremely large breeding population that has accumulated many recombination events, which is desirable for genome-wide association studies. We therefore examined the degree of genome-wide variation within CD-1 mice to investigate their suitability for genetic studies. The CD-1 mouse genome displays patterns of linkage disequilibrium and heterogeneity similar to wild-caught mice. Population substructure and phenotypic differences were observed among CD-1 mice obtained from different breeding facilities. Differences in genetic variation among CD-1 mice from distinct facilities were similar to genetic differences detected between closely related human populations, consistent with a founder effect. This first large-scale genetic analysis of the outbred CD-1 mouse strain provides important considerations for the design and analysis of genetic studies in CD-1 mice.

## Introduction

CD-1 mice are an inexpensive, robust and readily available outbred population commonly used in toxicology and cancer research [1], [2], [3]. They have also been widely used for mouse transgenesis experiments, principally due to efficient breeding and large litter sizes. Although spontaneous mutations have arisen in CD-1 mice, very few have been mapped. The mutations that have been identified in CD-1 mice involved commonly used inbred mouse mapping strategies, including complementation testing of candidate genes or mapping by outcrossing to a genetically characterized inbred strain [4], [5]. However, CD-1 mice are applicable to a broad range of genetic studies. While many large-scale examinations of the genetic architecture of inbred mice have been completed [6], [7], [8], [9], [10], [11], no comparable evaluations of commercially available outbred strains, including CD-1 mice, have been reported. This lack of genome-wide evaluation has created a significant obstacle to realizing the utility of CD-1 mice for genetic research.

Surprisingly little is known about the degree of heterogeneity that has survived within the various strains of outbred laboratory mice during their extended period of captive breeding, despite the reasonably well-documented historical relationship among both inbred and outbred laboratory mice [3], [12]. In fact, warnings against the use of commercially available outbred mice in genetic research have appeared in the literature due to the presumption that genetic variation within outbred mice cannot be easily maintained and may be highly variable across breeders and over time [13], [14], [15]. These warnings question whether outbred mice are actually genetically diverse mouse populations. Most outbred stocks are derived from a small number of mice that were imported to the US by Clara J. Lynch in 1926 and are collectively known as Swiss mice [3]. Reports examining allelic variation affecting enzymatic activity in outbred CD-1 mice and its inbred derivatives concluded that random fixation, but not inbreeding or population bottlenecks, accounted for slight losses in genetic variation among outbred mouse colonies [1], [2]. Although outbred mice are commonly cited as models for outbred human populations [1], [2], [3], based on their histories, it is more likely that outbred mice reflect human founder populations rather than outbred human populations. Large-scale evaluation of the genetic variation within commercially available outbred mice would resolve whether these mice are outbred and how they compare to human populations.

Currently, the mouse quantitative trait loci (QTL) mapping community is focused on creating novel inbred-based mouse populations to increase recombination events and thereby reduce linkage disequilibrium (LD) to facilitate fine-mapping studies. This initiative has culminated in the ongoing Collaborative Cross (CC) [16], [17], [18], [19], [20]. Several existing mouse populations, including outbred and wild-caught mice, also represent attractive alternatives to inbred mice for association mapping. In wild-caught mice from Arizona, LD decays at a rate favorable for high resolution association studies [21]. However, many standard phenotyping procedures for laboratory mice are extremely challenging to perform in wild-derived inbred strains [18], [22], and are likely to prove to be similarly difficult to carry out in wild-caught mice. In contrast, outbred mice are readily available, relatively inexpensive and standard phenotyping protocols can be used without modification. Currently, MF1 is the only outbred strain that has been utilized for QTL mapping [23], [24]. CD-1 mice have been used to examine the inherent genetic variability among common laboratory phenotypes such as discrimination learning [25], lever pressing, and locomotion [26], as well as phenotypic traits that model features of common complex human phenotypes, including stress reactivity [27], lithium response [28], and ingestion [29], [30], [31]. Despite this extensive, documented phenotypic variation, only one QTL has been reported in CD-1 mice and this was identified through a candidate gene approach [32]. The usefulness of CD-1 mice for identifying QTL has not been thoroughly examined.

In this report we present the first characterization of genetic variation and population structure of outbred CD-1 laboratory mice. Charles River Laboratories (CRL) has genotyped microsatellite markers (“Max-Bax”) to confirm that genetic variation in CD-1 mice has been maintained within their breeding facilities. However, this information is not extensive, not routinely collected, nor is it publicly available (CRL, personal communication). The initial sequencing of the mouse genome [7] and the development of high-throughput genotyping technologies have facilitated the discovery of over 8 million single nucleotide polymorphisms (SNPs) among classical inbred strains and provided relatively inexpensive, dense genotyping [10], [33]. SNPs identified between pairs of inbred strains have also been relatively successfully when genotyped in both MF-1 and wild-caught mice [21], [23], [24]. Here we used two relatively dense genome-wide sets of SNPs to investigate the genetic structure of >200 non-sibling CD-1 mice to better understand the LD and population structure in CD-1 mice. We also determined the genetic similarity of CD-1 to other Swiss mice and to inbred strains that will make up the CC. Finally, we evaluated the population substructure that exists among CD-1 mice from different production facilities maintained by a commercial vendor (CRL) and compare this substructure to human populations. We conclude that CD-1 mice provide a powerful and attractive complementary, readily available and inexpensive resource to currently used mouse populations for genetic studies.

## Results

### Genetic Variation in CD-1 Mice

We began investigating the genetic diversity of CD-1 outbred laboratory mice in a pilot study in which we evaluated three small genome-wide marker panels in a small number of unrelated CD-1 mice (**Table 1**). First, we genotyped the Max-Bax microsatellite panel available through CRL that contains 110 markers and determined that 79% of the markers were polymorphic. Second, we genotyped 394 autosomal SNPs (Panel MB1) [34] and determined that 75% of the markers were polymorphic. Third, we genotyped 768 SNPs (Panel MB2) [35] and determined that 51% of the markers were polymorphic. The low informativeness of the MB2 panel is likely the result of marker selection. Furthermore, none of these panels was sufficiently dense to evaluate LD among CD-1 mice.

**Table 1:** Marker panels genotyped in CD-1 mice.

Panel	Marker Type	# Markers	# Genotypes	# Polymorphic	Inter-polymorphic (Mb)
Max-Bax	microsatellite	110	108	85	24
MB1	SNP	394	247	186	11.4
MB2	SNP	768	757	388	6.5
MDL	SNP	1,449	1,311	935	2.7
IP	SNP	8,470	2,261	1,688	1.5

To more comprehensively assess CD-1 mice, we next examined two large cohorts of CD-1 outbred laboratory mice using larger SNP panels (**Table 1**). Cohort 1 consisted of tail DNA from 173 male (n = 83) and female (n = 90) CD-1 mice from the North Carolina (NC) breeding facility of CRL. Cohort 1 mice were genotyped using the Illumina Medium Density Linkage panel (MDL). Cohort 2 consisted of 72 male mice from three CRL breeding facilities (North Carolina (NC), Michigan (MI) and New York (NY)). Cohort 2 mice were genotyped using a custom designed SNP panel (IP). All SNPs were originally ascertained in inbred laboratory mice. Combined, these panels assessed 3,572 SNPs across the genome and were the basis of all further analyses.

**Figure 1:**
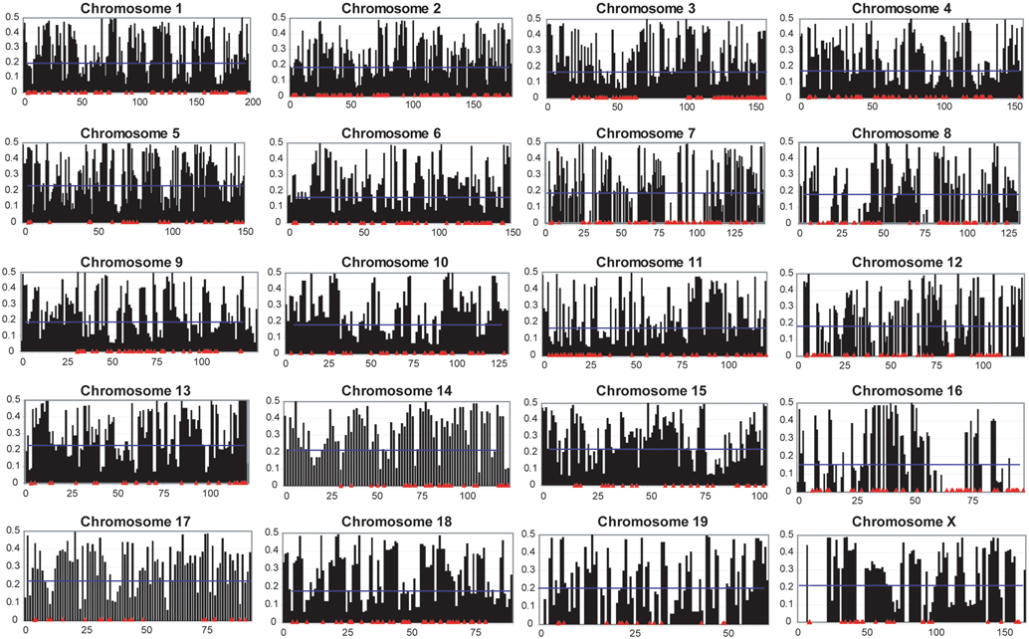
Chromosomal distribution of MAFs. For each chromosome, chromosomal position in Mb is shown on the X-axis, and MAF is plotted on the Y-axis. MAF values for individual SNPs are shown in black, the blue line represents mean MAF for each chromosome, and locations of monomorphic SNPs are in red.

The minor allele frequencies (MAF) of the SNPs from both Cohort 1 and 2 are summarized in **Figure 1** **and Figure S1**. Considering each autosome independently, the mean MAF ranges from 0.16 to 0.24 (s.d.±0.02), with a mean MAF of 0.31 for chromosome X and a mean genome-wide MAF of 0.28. The density of monomorphic SNPs ranges from 1.6 to 4.6 Mb per SNP (s.d.±0.79) on each autosome and is 5.9 Mb per SNP on chromosome X. More extensive analysis focused on common SNPs (MAF>0.05) present in CD-1 mice. Among the SNPs genotyped in this study, 73% are polymorphic and 68% have a MAF>0.05 in CD-1 mice. The mean density of polymorphic SNPs is one SNP per Mb. The density of common SNPs ranges from 659 kb to 1.4 Mb (s.d.±0.19) per SNP per autosome and is 1.6 Mb per SNP on chromosome X, with a mean genome-wide density of 1 Mb per SNP. Several chromosomes harbor regions with few informative SNPs. Since SNP selection is certain to influence these results, additional analysis is required to definitively conclude which regions within the CD-1 genome are not amenable for gene identification due to possible allele fixation in the CD-1 population. On average, 25% of the genotyped SNPs are polymorphic within any individual CD-1 mouse.

**Figure 2:**
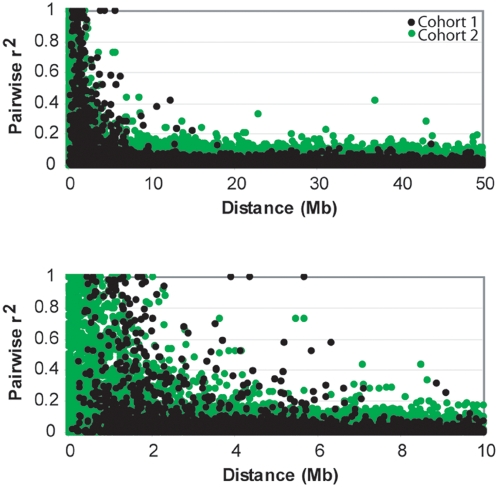
LD between ∼16,000 pairs of SNPs in CD-1 mice. The r^2^ measure of LD is shown as a function of physical distance for all common SNP pairs (top) and for common SNP pairs with an inter-SNP interval of ≤10 Mb (bottom) within Cohorts 1 and 2.

To examine the pattern of LD observed in CD-1 mice, we calculated the square correlation coefficient (r^2^) between pairs of SNPs genotyped in Cohorts 1 and 2 independently using Haploview [36]. The decay of LD between common SNP pairs as a function of physical distance is shown in **Figure 2**. The mean pair-wise LD for autosomal SNPs genotyped in Cohort 1 is r^2^ = 0.02 (r^2^ = 0.06 for chromosome X SNP pairs). The mean pair-wise LD for autosomal SNPs genotyped in Cohort 2 is r^2^ = 0.04. The majority (98%) of SNP pairs ≤10 Mb apart are in weak LD (r^2^≤0.30; **Figure S2**). In Cohort 1, among autosomal SNP pairs <1 Mb apart, the mean r^2^ = 0.30 (r^2^ = 0.69 for chromosome X SNP pairs). In Cohort 2, among autosomal SNP pairs <1 Mb apart, the mean r^2^ = 0.37. Though on average closely spaced SNPs (<1 Mb) were in low LD, strong LD (r^2^≥0.8) was observed over a physical distance ranging from 976 bp to 4.3 Mb with a mean distance of 474 kb among common SNP pairs. Since the two cohorts of CD-1 mice were genotyped independently, it is likely that strong LD exists between pairs of SNPs across the two cohorts that we were unable to capture in our analysis.

### Ancestry

**Figure 3:**
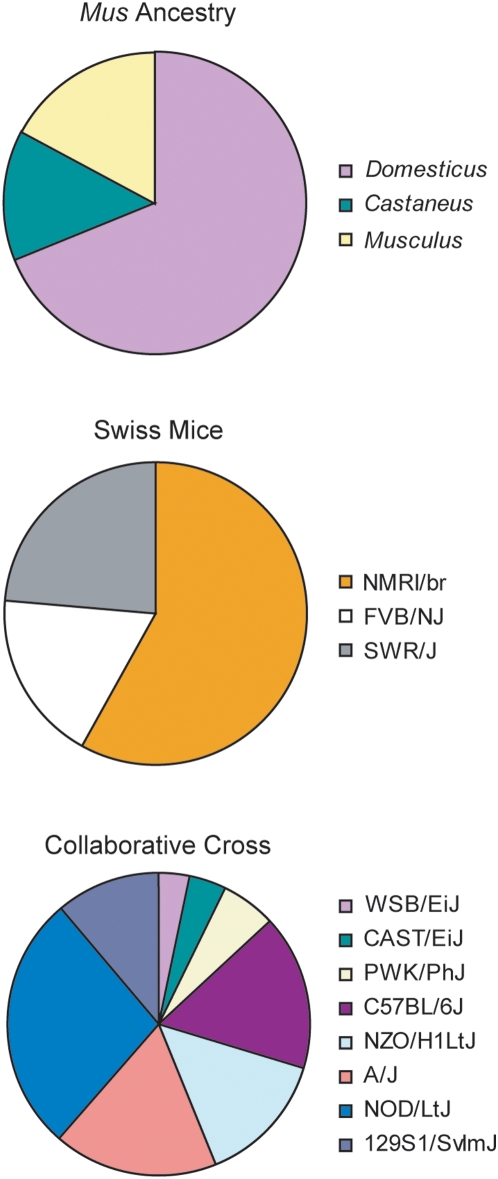
Inbred mouse contributions to CD-1 genomic variation as determined by *structure* analysis. Outbred CD-1 mice are represented by three pie charts that are partitioned into K colored segments to represent the CD-1 estimated membership K inbred subpopulations. Among wild-derived inbred strains (K = 3) representing the *Mus* subspecies, CD-1 mice are mostly *M. m. domesticus*. Among Swiss inbred mice (K = 3), CD-1 mice share the most genetic similarity with NMRI/br. CD-1 mice are 58% NMRI/br, 24% SWR/J and 18% FVB/NJ. Among CC inbred mice (K = 8), outbred CD-1 mice are most geneticly similar to NOD/LtJ (27%) and less similar to the other 7 strains (3% WSB/EiJ, 4% CAST/EiJ, 6% PWK/PhJ, 17% C57BL/6J, 14% NZO/H1LtJ, 18% A/J, and 11% 129S1/SvlmJ).

We next used the program *structure* [37], [38] to assess the genetic contribution of inbred mice to the CD-1 genome in three ways: (1) to confirm CD-1 mouse *Mus* subspecies ancestry, (2) to determine CD-1 mouse genetic relationship to other Swiss mice, and (3) to evaluate CD-1 mouse genetic relationship to parental strains of the CC (**Figure 3**).

The inclusion of three wild-derived inbred strains representing the *Mus* subspecies in Cohort 1 allowed us to better define the ancestry of CD-1 mice. Ancestry for 75% of the CD-1 genome could be assigned to the *M. m. domesticus* strain (WSB/EiJ), 19% to the *M. m. musculus* strain (PWD/PhJ), and 6% to the *M. m. castaneus* (CAST/EiJ) strain. Therefore, of the *Mus* subspecies, *domesticus* made the largest genetic contribution to the CD-1 genome, a finding consistent with the genealogical reports of outbred Swiss mice [3].

We also evaluated the genetic relationship between CD-1 mice and other Swiss-derived inbred strains, including FVB/NJ, NMRI/br and SWR/J, since these mice are all derived from recent common ancestors. We specified three populations (K = 3), using prior population information for the inbred strains, and partitioned the CD-1 mouse genome into its cognate Swiss inbred mouse makeup. CD-1 mice were most similar to the NMRI/br strain, followed by SWR/J, and finally FVB/NJ. We also evaluated the genetic relationship between CD-1 and ICR, a CD-1 strain maintained by another vendor (Harlan). When analyzed together, CD-1 and ICR mice were most consistent with a single population, though a two subpopulation model correctly placed individual mice into either the CD-1 or ICR population (Figure S3). As expected, comparison of ICR with the three Swiss inbred strains produced results similar to those observed for CD-1 mice. Not surprisingly, when we specified prior population information for the three Swiss inbred strains, and evaluated a four subpopulation model (K = 4) for the ICR and CD-1 oubred strains, ICR and CD-1 were consistent with one subpopulation rather than consisting of subpopulations of the Swiss inbred strains (data not shown).

Finally, we evaluated the genetic relationship between CD-1 mice and the CC parental strains (A/J, C57BL/6J, 129S1/SvImJ, NOD/LtJ, NZO/H1LtJ, CAST/EiJ, PWK/PhJ, and WSB/EiJ) [16], [17], [18], specifying eight populations (K = 8) in this analysis (Figure 3). We observed that CD-1 mice were most similar to the NOD/LtJ strain, with moderate similarity to the other 7 strains. CD-1 mice were least similar to the non-*domesticus* wild-derived strains CAST/EiJ and PWK/PhJ.

### Population structure

Population stratification due to allele frequency differences between cases and controls is well known to cause false positive associations in human disease studies [39], [40]. Genetic association studies in inbred mice are similarly confounded by the problem of spurious associations due to both population structure and genetic relatedness [41], [42]. We considered the evidence for detecting unknown familial relationships, or cryptic relatedness, among individual CD-1 mice by assessing departures from Hardy-Weinberg equilibrium (HWE). We did not detect any significant HWE deviations, with an excess of homozygotes at only 3% of autosomal loci (α = 0.05) in CD-1 mice. We also used PLINK [43] to estimate the sharing of genetic information by estimating the inbreeding coefficient (F) per individual and identity by descent (IBD) between pairs of CD-1 mice. The mean F was 0.005 among CD-1 mice in Cohort 1 and 0.05 in Cohort 2, consistent with populations of unrelated CD-1 mice, with a few individual exceptions (**Figure S4**). Further, the proportion of monomorphic SNPs was highly correlated with F (R^2^ = 0.99) for each animal. IBD estimates also provided evidence that the CD-1 mice in this study were unrelated with pi-hat <0.25 for 99% and 98% pairs of CD-1 mice within Cohorts 1 and 2, respectively. The CD-1 mice in Cohort 2 appeared to be more closely related as compared with the mice in Cohort 1, though this result may be an artifact of the SNP selection between the two groups.

**Figure 4:**
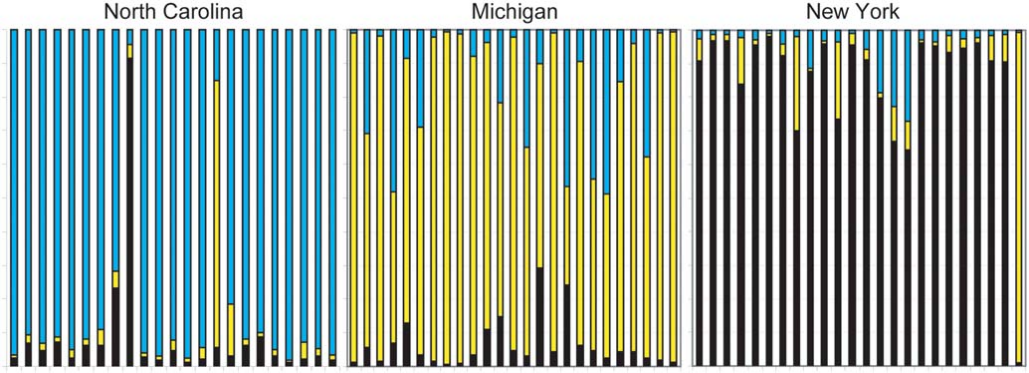
Estimated population structure among CD-1 mice. Each Cohort 2 individual is represented by a vertical bar that is partitioned into 3 colored segments (Black–NY, Yellow–MI, Blue–NC) to represent the individual's subpopulation membership. Breeding facilities are labeled above the panels. These results were consistent over 5 independent runs of the program.

CD-1 mice are expected to be a relatively genetically homogeneous population since they originate from a small number of founder mice [3]. However, CD-1 colonies are maintained at multiple breeding facilities. Therefore, we evaluated the possibility that population substructure exists among unrelated mice from different CRL breeding facilities, which would be a significant concern when using CD-1 mice for genome-wide association studies (GWAS). As expected, independent *structure* analysis of Cohorts 1 and 2 determined that both groups were most consistent with a single population (**Figure S5**). However, the CD-1 mice comprising Cohort 2 were deliberately obtained from three different breeding facilities, potentially representing population isolates. Using *structure*, we specified three populations (K = 3) and clearly partitioned the CD-1 mice into three subpopulations, consistent with their origins from three different breeding facilities (**Figure 4**). Individual mice obtained from the same breeding facility were almost always most similar to each other with the exception of a few outliers that were more similar to mice from another facility. CD-1 mice from the NY facility were most similar to each other, whereas the mice from the MI facility were the most diverse. In agreement with the *structure* data, multidimensional scaling (MDS) of pair-wise identity by state (IBS) sharing data also clustered the CD-1 mice according to breeding facility with the exception of a few outliers (**Figure S6**). Since Cohorts 1 and 2 were genotyped on different platforms that overlapped by only 17 SNPs (**Table S1**), we were unable to perform *structure* analysis on a combined data set. However, allele frequency comparisons among these small numbers of SNPs showed that Cohort 1 mice were most highly correlated with the NC group from Cohort 2 (**Table S2**).

**Figure 5:**
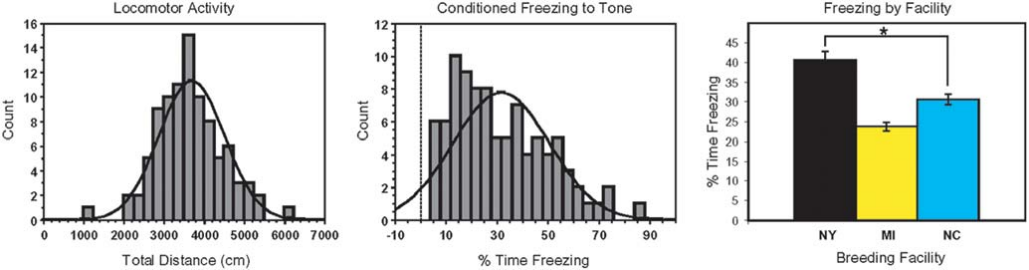
Behavioral phenotypes observed in Cohort 2. Frequency histograms for locomotor activity in an open field and the percent time spent freezing in response to a tone (freezing-to-tone) that was previously paired with a footshock are shown. The average freezing-to-tone scores (±SEM) for CD-1 mice grouped according facility of origin (Black–NY, Yellow–MI, Blue–NC) are also shown. *P = 0.001.

Population stratification can confound association studies particularly when the phenotype that is examined differs among populations. We therefore investigated whether genetically distinct populations of outbred CD-1 mice might be phenotypically different across the three separate breeding facilities. We examined two behavioral traits in CD-1 mice: locomotor activity and conditioned freezing to tone (**Figure 5**). Locomotor activity was normally distributed among CD-1 mice, while skewing was observed for conditioned freezing to tone. When breeding facility was used as a between subjects factor, we detected a significant difference in conditioned freezing to tone among mice from the three facilities. Since so few mice were genotyped, we did not have sufficient statistical power to perform GWAS mapping for these traits. However, markers that are also correlated with location would tend to show inflated association with conditioned freezing to tone due to population structure; this would produce excessive false positive errors. Our small pilot analysis cannot distinguish whether these behavior differences are due to genetic or environmental factors. Regardless, our identification of population substructure is of particular importance and demonstrates that CD-1 mice are best regarded as multiple related populations versus a single unified population.

**Figure 6:**
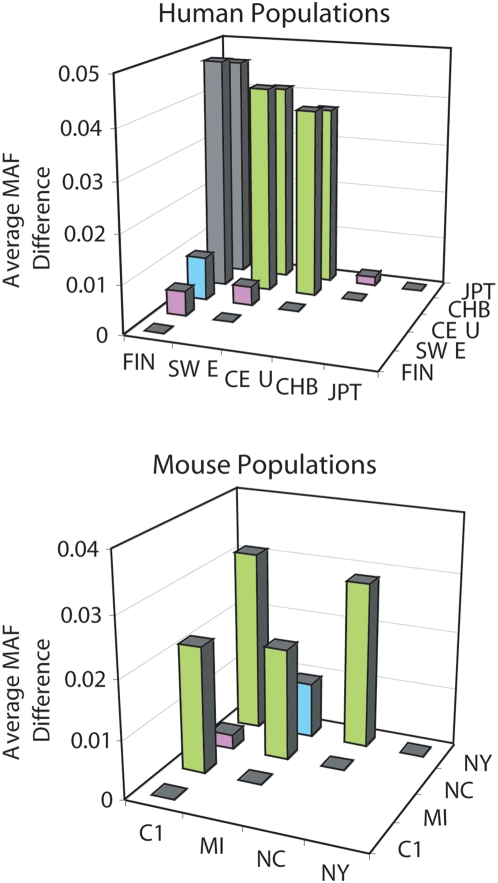
Human and mouse population comparisons. Pair-wise comparisons of average MAF differences among human founder (FIN–Finnish, SWE–Swedish) and HapMap (CEU–CEPH, CHB–Chinese, JPT–Japan) populations and CD-1 mouse populations (C1–Cohort 1, MI–Michigan, NC–North Carolina, NY–New York). Colors represent similar differences observed between pairs of human and mouse populations (purple<0.01, blue = 0.01, green>0.02).

To examine the possibility that outbred CD-1 mice model human founder populations, we compared the average MAF differences observed among subpopulations of CD-1 mice to a previous study of human populations (**Figure 6**). Among five human populations (Finnish, Swedish, CEPH, Japanese and Chinese), the MAF averaged for 34 autosomal SNPs ranged from 0.39 (Finnish) to 0.44 (Chinese). Among four CD-1 mouse populations (Cohort 1, NC, MI and NY), the MAF averaged for 16 SNPs ranged from 0.20 (NY) to 0.23 (Cohort 1). The average MAF differences (<1%) observed between three pairs of human populations (Finnish and Swedish, CEPH and Swedish or Chinese and Japanese (Asian)) was comparable to the average MAF differences detected between the two groups of CD-1 mice obtained from the same facility (Cohort 1 and NC). CD-1 mice from Cohort 1 or NC compared to MI (2%) or NY (3%) were nearly as distinct as European (Swedish or CEPH) and Asian populations (4%). MI and NY mouse populations differed similarly to Finnish and CEPH (1%). These data are only suggestive due to the small number of CD-1 SNPs available to compare differences among mouse populations.

## Discussion

The major conclusions in this study pertain to the suitability of CD-1 mice for mouse genetic studies. CD-1 mice are a widely used, inexpensive and readily available outbred mouse population that has not been previously systematically genetically characterized. We have determined that CD-1 mice are polymorphic at a significant number of loci, are reasonably outbred, and have a complex genetic history similar to a human founder population. Furthermore, though population substructure may confound mapping results in these mice when it is unrecognized, it could also be further exploited to identify phenotypic or genetic subgroups for genetic mapping. Thus, we have shown that CD-1 mice represent an excellent genetic mapping resource of broad utility to the mouse genetics community.

We have also shown that SNPs ascertained in inbred strains can successfully capture the genetic variation in outbred CD-1 mice. The SNP panels utilized in this study were not selected for their informativeness in CD-1 mice, creating biases in SNP selection that have affected our results. Despite the limitation of SNP selection, we have shown that common SNPs occur within CD-1 mice at a rate comparable to the rate observed within wild-caught mice [21]. Although the genotyped panels of SNPs were not dense enough to support conclusive evaluation of LD structure in CD-1 mice, our data provide an estimation of LD structure sufficient to evaluate suitability for genetic studies. CD-1 mice will provide high-resolution mapping that requires dense genotyping similar to the heterogeneous stock [44]. A newly designed JAX Mouse Diversity Genotyping Array that will permit genotyping of >600,000 SNPs (http://jaxservices.jax.org/mdsnp) will provide a genotyping platform that does not require careful SNP selection and will capture even more genetic variation. This data will allow for construction of haplotype maps and facilitate the resolution of QTL in all heterogeneous mice, including outbred CD-1 mice.

Among inbred laboratory strains, the inbred Swiss mice are genealogically most closely related to outbred CD-1 mice [3], [12]. Among the inbred Swiss strains available for comparison in this study, NMRI/br is the most genetically similar to CD-1 mice. NMRI/br has been maintained as an inbred line since the late 1970s, which is the most recent inbred of the Swiss mice examined in this study. Our data suggest that NMRI/br mice have retained much of the genetic diversity of CD-1, its most recent common ancestor strain. However, CD-1 mice are more genetically similar to SWR/J, a line that has been inbred since 1926, than they are to FVB/NJ, which has been maintained as an inbred line only since the 1970s following many rounds of inbreeding and interbreeding. These results suggest that bottlenecks created during the generation of inbred mice differentially capture the genetic diversity observed within the parental outbred strain resulting in either retention or loss of the original variation. Importantly, we have shown that CD-1 and ICR mice, which derived from the same outbred stock but have been isolated and maintained by two different vendors, were correctly partitioned into two subpopulations.

The CC was initiated to generate a reproducible mouse population with an accumulation of recombination events to facilitate genetic studies [16]. In comparisons between CD-1 mice and the eight inbred parental CC strains, we have shown that CD-1 is most genetically similar to NOD/LtJ. This result is not surprising since the NOD/LtJ strain originated from Swiss derived ICR mice and we demonstrated that CD-1 and ICR mice are genetically very similar. Interestingly, although the NZO/H1LtJ strain is also derived from ICR mice, CD-1 mice are about equally similar to the related inbred strains A/J and C57BL/6J as they are to the NZO/H1LtJ strain. Overall, CD-1 mice are less similar to the wild-derived inbred strains used in the CC. The relationship of CD-1 to the CC is important to consider since both populations have complementary strengths and weaknesses for fine-scale mapping.

Our studies have revealed population substructure among CD-1 mice originating from more than one facility. Remarkably, in our analysis the majority of CD-1 mice were grouped with their originating CRL facility. Two explanations are possible for the few mice that were assigned to an alternative subpopulation as compared to their originating CRL breeding facility. The first possibility is that the mice/DNA samples were mislabeled at some point subsequent to their shipment from CRL. Alternatively, outlier mice may have indeed originated from the indicated facility. While no records were available, CRL confirmed that they periodically transfer breeders from one facility to another to maintain genetic diversity among all CD-1 production facilities (CRL, personal communication). Thus, the apparent outliers may reflect descendents of mice that were recently transferred.

We provide examples of routinely collected mouse phenotypes that are normally distributed within outbred CD-1 mice. We also observed significant differences in phenotypes for mice obtained from different facilities. The small numbers of mice phenotyped prevented GWAS analysis, but we note that phenotypic divisions and population substructure could substantially affect the results of GWAS causing spurious associations. We have demonstrated that these differences are easily detectable and suggest that they could further be leveraged to facilitate genetic studies (e.g. CD-1 mice from MI are more heterogeneous than CD-1 mice from NC and NY). We suggest that the extensive phenotypic data available for CD-1 mice due to their common use in toxicology and cancer research be extended to association mapping of these phenotypes.

Finally, our CD-1 mouse data are consistent with recent reports describing the correlation between fine-scale genetic substructure and geographical locations within human populations [45], [46]. Comparison of the Swedish and Finnish founder populations to the three genetically distinct HapMap populations clusters the Swedish and Finnish populations with European CEPH due to the origin of Scandinavian populations. When the closely related CEPH, Swedish and Finnish populations are compared, multidimensional scaling separates the CEPH, Swedish and Finnish subisolates [47]. CD-1 mouse populations are overall genetically less diverse than Swedish, Finnish, or HapMap populations, but they are similarly separated into subpopulations when grouped according to facility of origin. Comparison between populations of CD-1 mice that originated from the same breeding facility revealed differences in genetic diversity similar to that observed between Swedish and Finnish or Chinese and Japanese human populations. Comparison between CD-1 mice from the most homogeneous facility (NY) with mice from the most heterogeneous facility (MI) revealed population differences similar to those observed between Finnish and CEPH human populations. Surprisingly, CD-1 mice from NC and NY were nearly as different as European and Asian human populations and should be explored further using a larger dataset. Our data suggest that the genetic variation observed among outbred CD-1 laboratory mice is consistent with CD-1 mice as an outbred population that is less diverse than human populations, but with genetic variation between populations that is similar to closely related human populations.

### Conclusions

In a large scale analysis of the outbred CD-1 mouse strain, we have determined the nature and extent of genetic variation within this previously genetically uncharacterized outbred mouse strain. We demonstrate that CD-1 mice have a suitable genetic structure to support high resolution mapping of both quantitative and qualitative traits. CD-1 mice thus provide a complementary, readily available and inexpensive resource to inbred mouse mapping populations.

## Materials and Methods

### Sample collection

All aspects of the study were approved by the Animal Care and Use Committee of the University of Chicago and were performed in the accordance with institutional policy and National Institutes of Health guidelines governing the humane treatment of vertebrate animals. Typical mouse orders contain many closely related (sibling) animals. We requested unrelated (non-sibling) CD-1 mice in order to maximize the genetic diversity within our sample. Individual tails from 173 CD-1 mice were obtained from the CRL breeding facility in North Carolina. An additional 96 live male CD-1 mice were obtained from the CRL breeding facilities in NC (n = 33), MI (n = 33), and NY (n = 33). Current breeding population size by facility as provided by CRL: MI: 2370 females in polygamous breeding per week; NY: 2 colonies 648 and 342 females in polygamous breeding per week; and RI: 1000 females in polygamous breeding per week. Pregnant females are pulled weekly prior to parturition and are returned to the breeding pool in about 6–8 weeks. DNA was extracted from CD-1 tails using PureGene (Gentra Systems). DNA for 4 wild-derived inbred strains (CAST/EiJ, MOLF/EiJ, PWD/PhJ, WSB/EiJ) and 1 inbred strain (FVB/NJ) was purchased from the Jackson Laboratory Mouse DNA Resource.

### Genotyping mice

Genome-wide sets of SNPs were genotyped for 245 CD-1 mice and five inbred strains (CAST/EiJ, MOLF/EiJ, PWD/PhJ, WSB/EiJ and FVB/NJ). The genotyping of 173 CD-1 mice (Cohort 1) and the five inbred strains was performed by the Genetic Resources Core Facility at Johns Hopkins University using the Illumina Medium Density Mouse Linkage Panel (MDL). This panel consists of 1,449 SNPs chosen from the Wellcome-CTC Mouse Strain SNP Genotype Set (http://www.well.ox.ac.uk/mouse/INBREDS), of which 1,311 (90%) were successful within the CD-1 mouse samples (**Dataset S1**). The success rate is 99% at each SNP and 98% for each individual. The genotyping of 72 CD-1 mice (Cohort 2) was performed by the W.M. Keck Foundation Biotechnology Resource Laboratory at Yale University using a custom designed Illumina mouse genotyping panel (IP). This panel consists of 8,470 SNPs chosen from multiple sources, of which 5,510 (65%) were successful within the CD-1 mouse samples at an Illumina GC cutoff of ≥0.80. However, heterozygous genotype calls for SNPs outside the pseudoautosomal region on chromosome X in these male mice caused us to increase our calling stringency to an Illumina GC cutoff of ≥0.90. Thus, 2,261 (26%) SNPs were confidently called within the CD-1 mouse samples (**Dataset S1**). The success rate is 99% for each SNP and for each individual. The overlap between the two SNP panels utilized in this study consisted of only 17 SNPs.

### Statistical analyses

Genotypic data were analyzed using Haploview v4.0 (http://www.broad.mit.edu/mpg/haploview/) to calculate allele frequencies and pairwise LD (r^2^). PLINK v1.02 (http://pngu.mgh.harvard.edu/~purcell/plink/) was used to calculate genotype frequencies, Hardy-Weinberg equilibrium, inbreeding coefficient (F), prune autosomal SNPs in strong LD (r^2^≥0.8), calculate IBD/IBS estimates, and perform multidimensional scaling. The Cohorts 1 and 2 SNP datasets were analyzed independently due to the paucity of overlapping SNPs and presented as a combined 3,572 SNP dataset, except where indicated. Cohort 1 (n = 173) genotypic data were used to evaluate monomorphic SNPs (n = 376). Cohort 1 genotypic data for female CD-1 mice (n = 90) were used to evaluate LD along the X chromosome.

### Publicly available mouse SNP data

Genotypes for 10 common inbred strains (129S1/SvlmJ, ARK/J, BALB/cJ, C3H/H3J, C57BL/6J, CBA/J, DBA/2J, FVB/NJ, NOD/LtJ, and SJL/J) from the MDL panel were downloaded from Illumina (http://www.illumina.com/downloads/Mouse_MD_Linkage_Inbred_Genotypes.pdf). Genotypes for inbred NMRI/br, SWR/J, A/J, NZO/H1LtJ, PWK/PhJ and outbred ICR (n = 9) were downloaded from the Wellcome-CTC Mouse Strain SNP Genotype Set (http://www.well.ox.ac.uk/mouse/INBREDS). After removing SNPs without genotypes (n = 7) and A/T or G/C SNPs for which homozygous allele assignment could not easily be determined in inbred mice (n = 146), genotypes for 1,157 (88%) were available for comparison with the 1,311 Cohort 1 SNP genotypes. Additional heterozygous genotypes (n = 117–128) at A/T or G/C SNPs in the outbred ICR mice were included for comparison with Cohort 1 CD-1 genotypes.

### Structure analysis

*Structure* 2.2.3 (http://pritch.bsd.uchicago.edu/structure.html) was used to infer the population substructure between (a) Cohort 1 CD-1 mice (n = 173) and three wild-inbred strains representing the *Mus* subspecies, (b) Cohort 1 CD-1 mice and other Swiss mice (three inbred and one outbred strain), (c) Cohort 1 CD-1 mice and eight inbred strains comprising the CC, and (d) among Cohort 2 CD-1 mice (n = 72) obtained from three different CRL facilities (NC, n = 23; MI, n = 25; NY, n = 24). Since LD within subpopulations is assumed, autosomal SNPs in strong LD were pruned to sets of 711 and 774 for Cohorts 1 and 2, respectively. Genotype data for each of the fourteen inbred strains was represented 250 times to generate fixed populations of inbred mice. The admixture model having three (a, b, d), four (b) or eight (d) subpopulations (K = 3, 4 or 8) was analyzed among CD-1 mice. Five independent runs of the program were made for each of the three analyses, with 5,000 burn-in and 10,000 subsequent steps.

### Behavioral testing of CD-1 mice

Locomotor activity was monitored for 20 minutes on a single day in mice that were naïve to the test environment, as described previously [48]. Subsequent to locomotor activity testing the same animals were tested for fear conditioning using standard 3-day protocol in which day 1 was used to associate the test chamber and a tone with shocks, day 2 assessed freezing behavior in response to the test chamber environment, and day 3 examined freezing in response to an altered context and subsequent freezing to presentation of the tone that had been paired with the shock on day 1 in the altered context.

### Human population substructure

To estimate the substructure observed among human populations comparable to the CD-1 mouse data, we computed the average MAF for a set of unlinked autosomal SNPs for Finnish, Swedish and HapMap CEPH, Han Chinese and Japanese populations [49]. The average MAF for CD-1 mouse populations was based on the small number of SNPs that overlapped between the two SNP panels after excluding one SNP (rs13480734) because the minor allele was not consistent across CD-1 subpopulations. We then calculated the average MAF difference for pair-wise comparisons of human (n = 20) and CD-1 mouse (n = 10) populations.

## Supporting Information

Figure S1 Coverage and MAF distribution of SNPs. Chromosomal position in Mb is shown. MAF values for individual SNPs are shown relative to chromosomal position. Regions that did not contain genotyped SNPs are shown in grey.
(5.15 MB TIF)

Figure S2 Distribution of SNP pair LD. Pairwise LD (r2) for ∼16,000 pairs of SNPs with MAF >0.05 is binned along the X-axis. Proportion of SNP pairs within each bin is shown along the Y-axis.
(0.27 MB TIF)

Figure S3 Population structure among Swiss mice. The result for each independent structure run is indicated with an open circle and the mean of 5 runs with a closed circle (top). The combined analysis of CD-1 and ICR mice is most consistent with a single population. However, despite the increase in likelihood for the two subpopulation model, the minimal variation between runs, together with the correct placement of individual mice into their CD-1 or ICR subpopulation (data not shown), supports the differentiation of CD-1 and ICR into 2 subpopulations. ICR mice are represented by a pie chart that is partitioned into 3 colored segments to represent the estimated relationship of ICR to 3 Swiss inbred populations (bottom). ICR mice are 55% NMRI/br, 25% SWR/J and 20% FVB/NJ.
(0.16 MB TIF)

Figure S4 Evidence against inbreeding among CD-1 mice. For each CD-1 mouse, the percent of monomorphic SNPs are shown along the X-axis and the inbreeding coefficient is shown along the Y-axis.
(0.14 MB TIF)

Figure S5 Population structure within CD-1 mice. The result for each independent structure run is indicated with an open circle and the mean of 5 runs with a closed circle. Both Cohorts 1 and 2 are most consistent with a single population. The considerable variability for the two and three subpopulation models within Cohort 1 and the increase in likelihood fails to support the differentiation of this population. However, despite the increase in likelihood for the two and three subpopulation models within Cohort 2, the minimal variability among runs for the subpopulation models supports differentiation of this otherwise genetically homogeneous population as compared to Cohort 1.
(0.31 MB TIF)

Figure S6 Results of multidimensional scaling in Cohort 2 CD-1 mice. The first two dimensions of variation in the subpopulations produce clusters of mice that are consistent with their originating breeding facility.
(0.21 MB TIF)

Table S1 MAF for 17 SNPs among CD-1 populations.
(0.14 MB DOC)

Table S2 MAF correlations between CD-1 subpopulations.
(0.05 MB DOC)

Dataset S1 Annotation for the genome-wide set of SNPs genotyped in CD-1 Cohorts 1 and 2. This file contains tab-delimited text in 3,573 rows and 7 columns.
(0.12 MB TXT)
