# Supplementary figures and images for: Genetic Variation and Population Substructure in Outbred CD-1 Mice: Implications for Genome-Wide Association Studies
Source: PLoS One. 2009 Mar 6;4(3):e4729. doi: 10.1371/journal.pone.0004729 (PMC2649211; doi:10.1371/journal.pone.0004729)

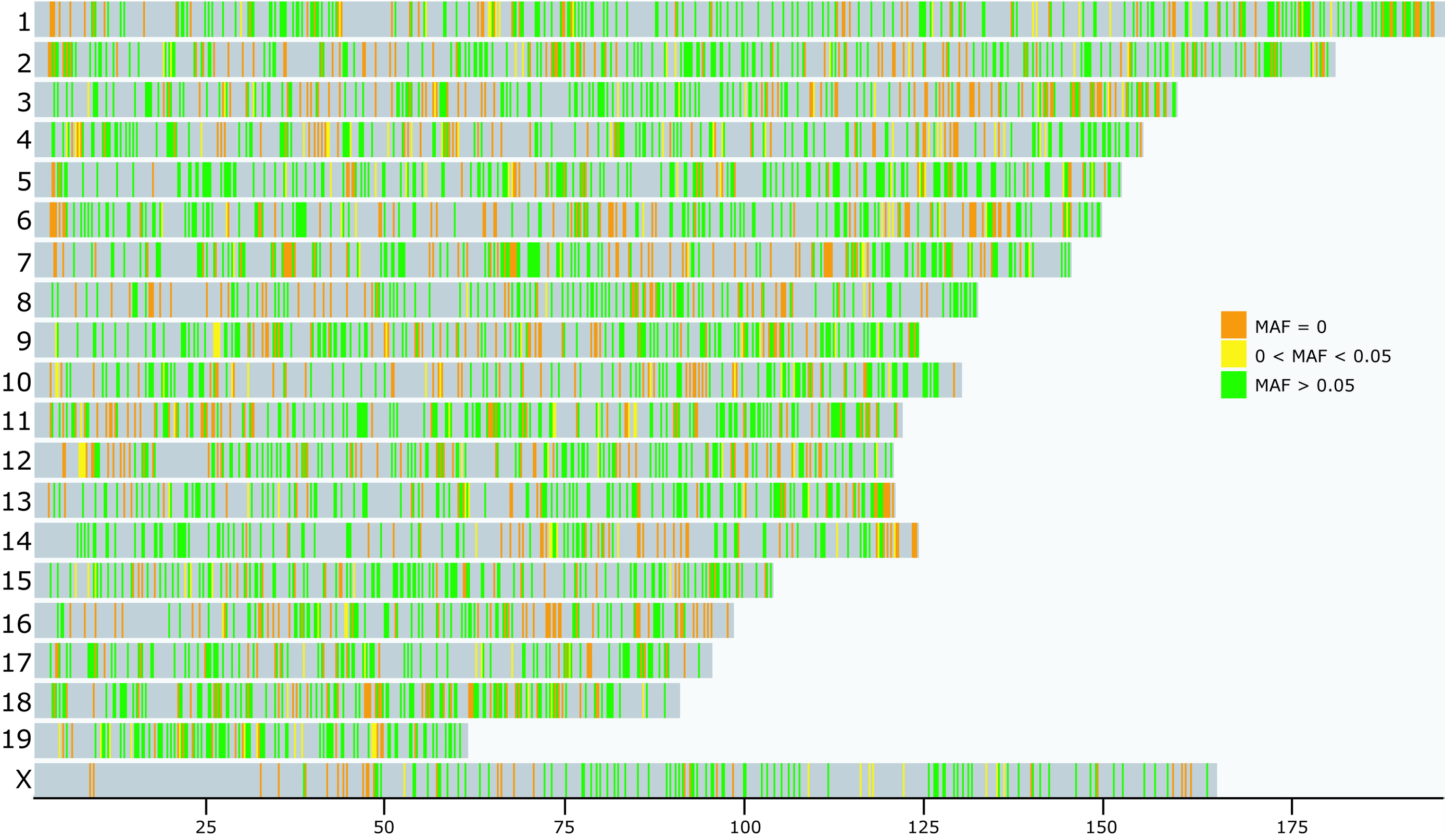

Supplement: Figure S1 — Coverage and MAF distribution of SNPs. Chromosomal position in Mb is shown. MAF values for individual SNPs are shown relative to chromosomal position. Regions that did not contain genotyped SNPs are shown in grey. (5.15 MB TIF) [file pone.0004729.s001.tif]

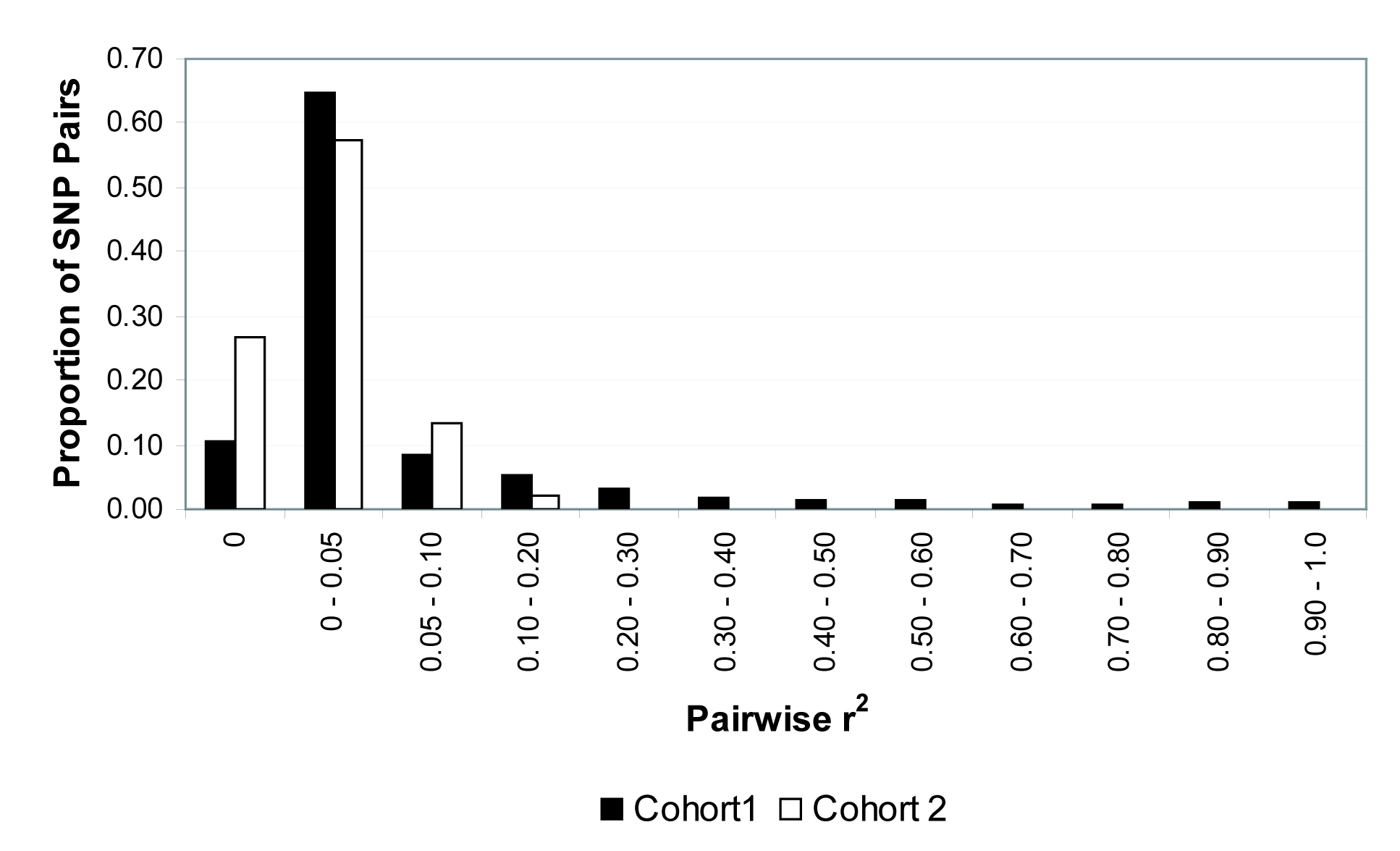

Supplement: Figure S2 — Distribution of SNP pair LD. Pairwise LD (r2) for ∼16,000 pairs of SNPs with MAF >0.05 is binned along the X-axis. Proportion of SNP pairs within each bin is shown along the Y-axis. (0.27 MB TIF) [file pone.0004729.s002.tif]

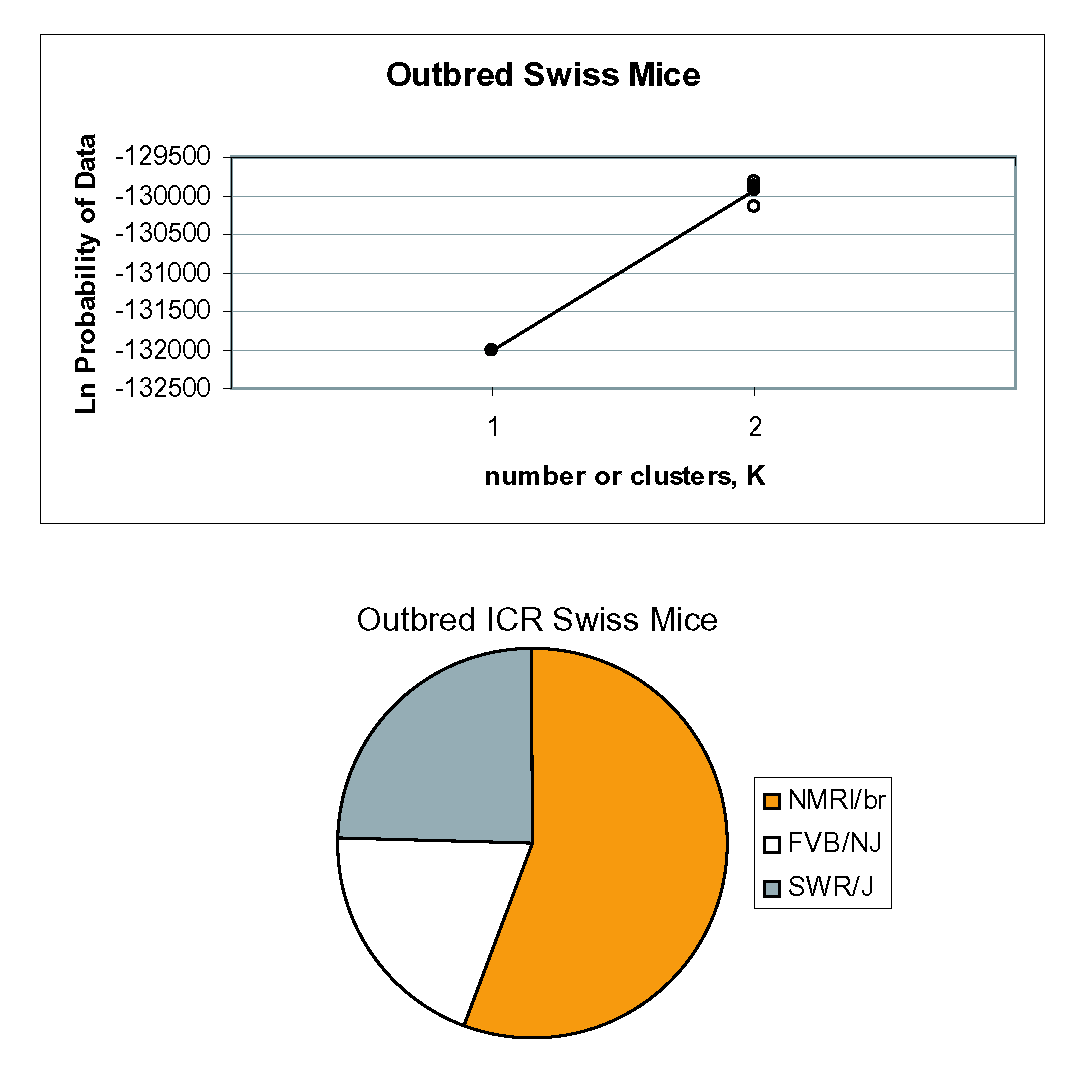

Supplement: Figure S3 — Population structure among Swiss mice. The result for each independent structure run is indicated with an open circle and the mean of 5 runs with a closed circle (top). The combined analysis of CD-1 and ICR mice is most consistent with a single population. However, despite the increase in likelihood for the two subpopulation model, the minimal variation between runs, together with the correct placement of individual mice into their CD-1 or ICR subpopulation (data not shown), supports the differentiation of CD-1 and ICR into 2 subpopulations. ICR mice are represented by a pie chart that is partitioned into 3 colored segments to represent the estimated relationship of ICR to 3 Swiss inbred populations (bottom). ICR mice are 55% NMRI/br, 25% SWR/J and 20% FVB/NJ. (0.16 MB TIF) [file pone.0004729.s003.tif]

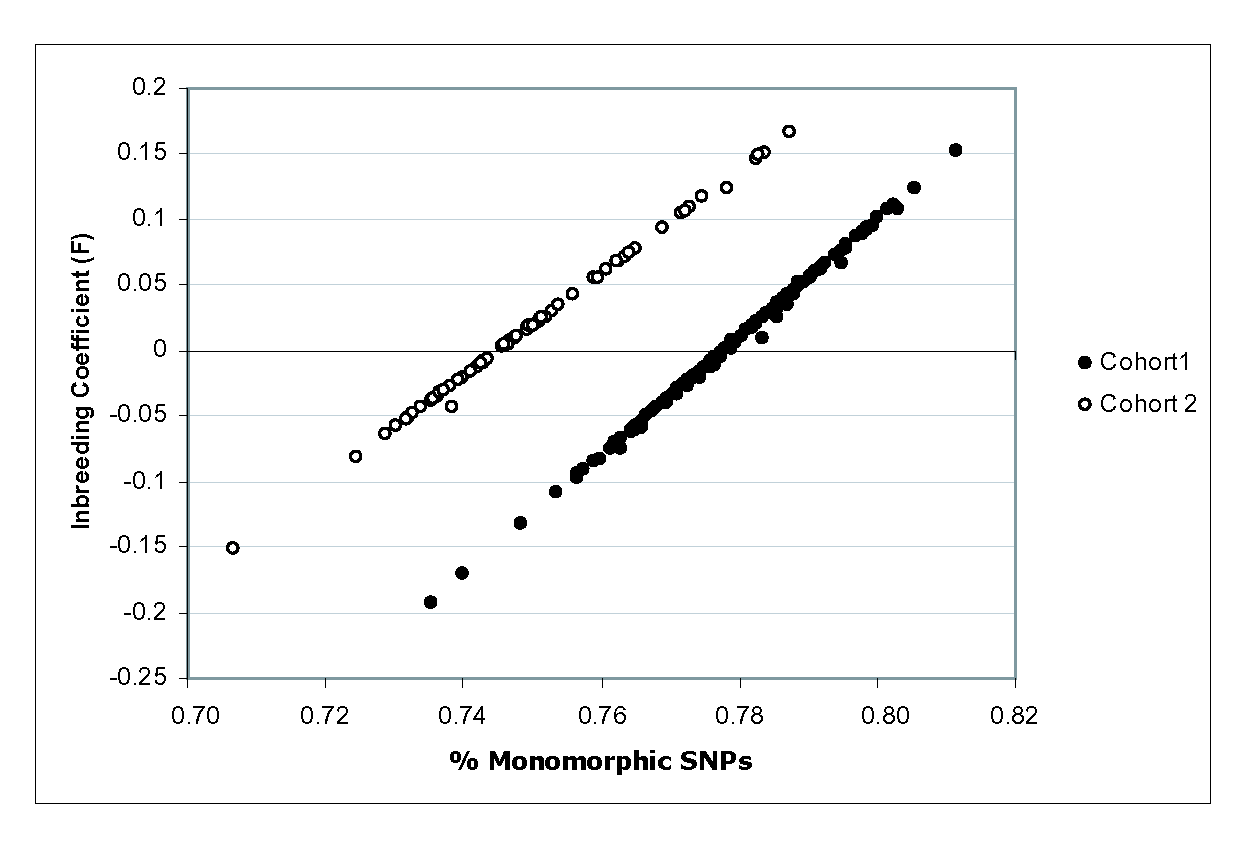

Supplement: Figure S4 — Evidence against inbreeding among CD-1 mice. For each CD-1 mouse, the percent of monomorphic SNPs are shown along the X-axis and the inbreeding coefficient is shown along the Y-axis. (0.14 MB TIF) [file pone.0004729.s004.tif]

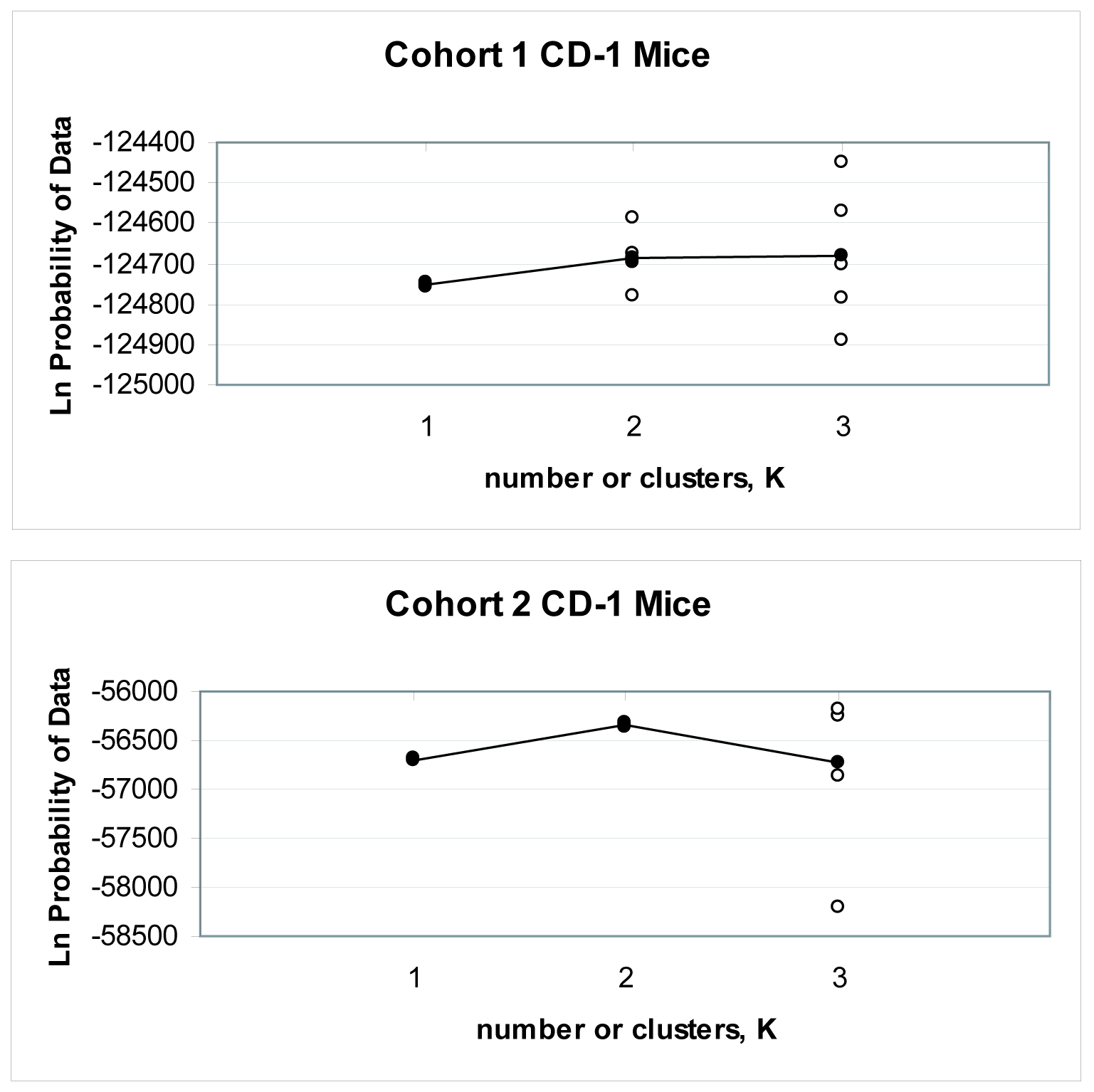

Supplement: Figure S5 — Population structure within CD-1 mice. The result for each independent structure run is indicated with an open circle and the mean of 5 runs with a closed circle. Both Cohorts 1 and 2 are most consistent with a single population. The considerable variability for the two and three subpopulation models within Cohort 1 and the increase in likelihood fails to support the differentiation of this population. However, despite the increase in likelihood for the two and three subpopulation models within Cohort 2, the minimal variability among runs for the subpopulation models supports differentiation of this otherwise genetically homogeneous population as compared to Cohort 1. (0.31 MB TIF) [file pone.0004729.s005.tif]

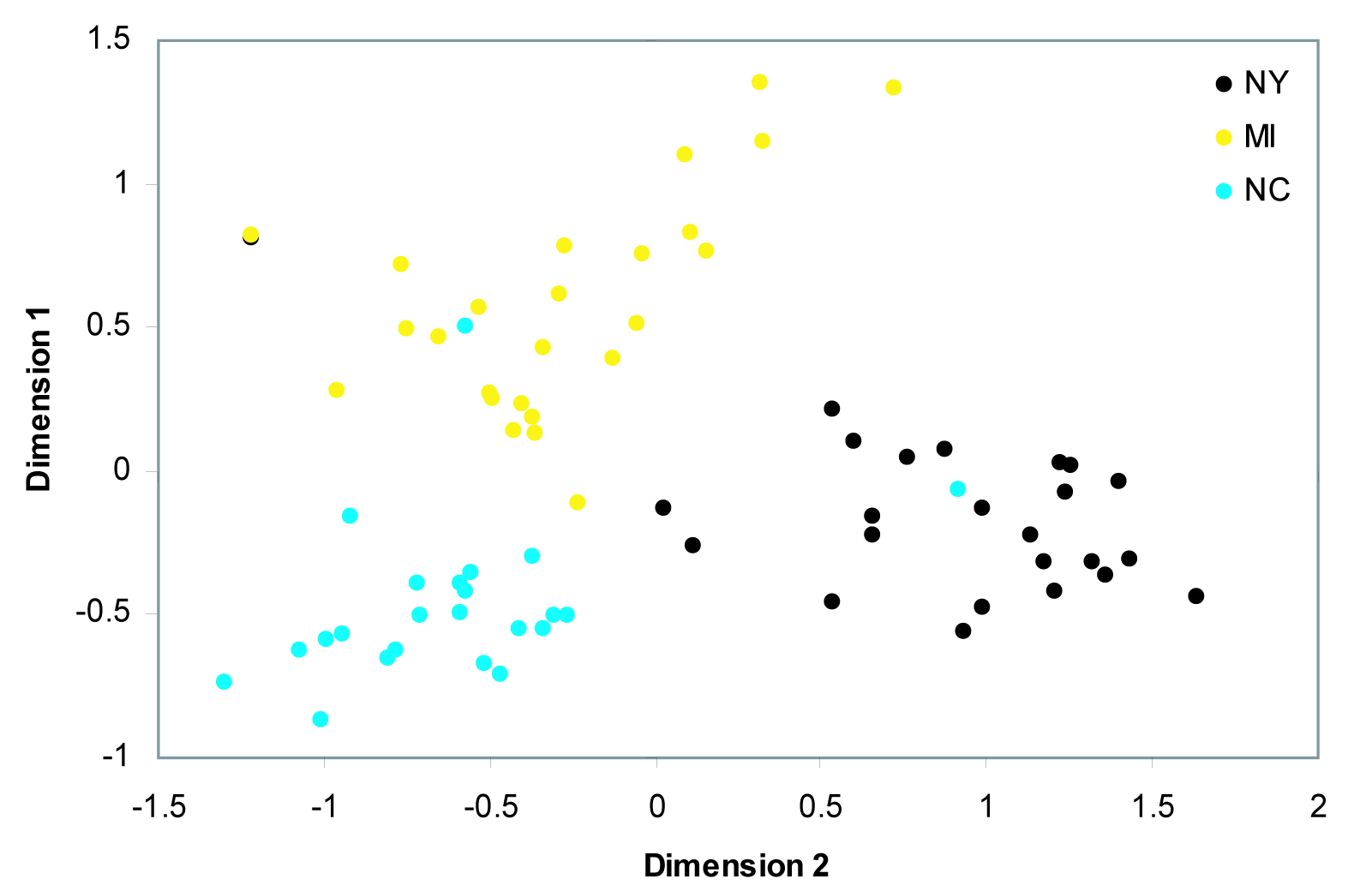

Supplement: Figure S6 — Results of multidimensional scaling in Cohort 2 CD-1 mice. The first two dimensions of variation in the subpopulations produce clusters of mice that are consistent with their originating breeding facility. (0.21 MB TIF) [file pone.0004729.s006.tif]
