# Supplementary material for: Genetic Variation and Population Substructure in Outbred CD-1 Mice: Implications for Genome-Wide Association Studies
Source: PLoS One. 2009 Mar 6;4(3):e4729. doi: 10.1371/journal.pone.0004729 (PMC2649211; doi:10.1371/journal.pone.0004729)
Supplement: Table S1 — MAF for 17 SNPs among CD-1 populations. (0.14 MB DOC) [file pone.0004729.s007.doc]

| SNP | Chr | Position^1^ | Alleles | Cohort 1 | | Cohort 2 | | Cohort 2 - NC | | Cohort 2 -MI | | Cohort 2 - NY | |
| --- | --- | --- | --- | --- | --- | --- | --- | --- | --- | --- | --- | --- | --- |
| rs6359983 | 2 | 6664321 | A/G | 0.21 | G | 0.22 | G | 0.28 | G | 0.16 | G | 0.22 | G |
| rs13477421 | 3 | 134179590 | A/A | 0.00 | A | 0.00 | A | 0.00 | A | 0.00 | A | 0.00 | A |
| CEL-5_45872918 | 5 | 47536005 | C/G | 0.33 | G | 0.32 | G | 0.26 | G | 0.36 | G | 0.33 | G |
| rs13478780 | 6 | 59452930 | G/C | 0.06 | C | 0.08 | C | 0.07 | C | 0.11 | C | 0.06 | C |
| rs13459097 | 6 | 84855960 | A/G | 0.07 | G | 0.14 | G | 0.07 | G | 0.23 | G | 0.13 | G |
| rs6329892 | 6 | 142377540 | G/A | 0.19 | A | 0.29 | A | 0.24 | A | 0.41 | A | 0.22 | A |
| rs13479619 | 8 | 15279348 | A/G | 0.36 | G | 0.19 | G | 0.37 | G | 0.07 | G | 0.15 | G |
| rs13480421 | 9 | 111703646 | C/G | 0.18 | G | 0.15 | G | 0.22 | G | 0.14 | G | 0.11 | G |
| rs6190748 | 10 | 55476940 | A/G | 0.01 | G | 0.07 | G | 0.02 | G | 0.00 | A* | 0.17 | G |
| rs13480734 | 10 | 101805079 | A/G | 0.48 | G | 0.47 | A* | 0.48 | G | 0.39 | G | 0.32 | A* |
| rs3661058 | 11 | 98735531 | G/A | 0.40 | A | 0.32 | A | 0.33 | A | 0.30 | A | 0.33 | A |
| CEL-12_84750094 | 12 | 90563853 | G/A | 0.17 | A | 0.19 | A | 0.20 | A | 0.07 | A | 0.28 | A |
| gnf13.038.133 | 13 | 40049739 | G/A | 0.20 | A | 0.21 | A | 0.17 | A | 0.21 | A | 0.24 | A |
| rs13481783 | 13 | 42851119 | G/A | 0.47 | A | 0.31 | A | 0.35 | A | 0.36 | A | 0.24 | A |
| rs13483183 | 18 | 3516539 | G/A | 0.38 | A | 0.33 | A | 0.41 | A | 0.27 | A | 0.32 | A |
| CEL-18_60214752 | 18 | 59824531 | G/A | 0.32 | A | 0.31 | A | 0.26 | A | 0.46 | A | 0.22 | A |
| CEL-X_125736335 | X | 131075537 | T/A | 0.10 | A | 0.10 | A | 0.17 | A | 0.05 | A | 0.07 | A |

^1^UCSC Mouse Genome Browser February 2006 (NCBI Build 36)

*Minor allele opposes Cohort 1 minor allele

The average MAF among the 17 SNPs for each CD-1 population ranged from 0.20 to 0.23. The average deviation from the mean for MAF at each of the 17 SNPs ranged from 0 to 0.11. The greatest difference in allele frequencies among CD-1 populations was observed for rs6190748, which was monomorphic only in the MI population. The minor allele for another SNP (rs13480734) was A in all populations except in NY where G was the minor allele.
