# Supplementary material for: Genetic Variation and Population Substructure in Outbred CD-1 Mice: Implications for Genome-Wide Association Studies
Source: PLoS One. 2009 Mar 6;4(3):e4729. doi: 10.1371/journal.pone.0004729 (PMC2649211; doi:10.1371/journal.pone.0004729)
Supplement: Table S2 — MAF correlations between CD-1 subpopulations. (0.05 MB DOC) [file pone.0004729.s008.doc]

|  |  | 1-tailed *P*-value | | | | |
| --- | --- | --- | --- | --- | --- | --- |
|  |  | Cohort 1 | Cohort 2 | Cohort 2 - NC | Cohort 2 - MI | Cohort 2 - NY |
| Pearson's  correlation | Cohort 1 | - | 3E-07 | 6E-09 | 0.001 | 3E-04 |
|  | Cohort 2 | 0.907 | - | 1E-06 | 1E-05 | 4E-06 |
|  | Cohort 2 - NC | 0.944 | 0.882 | - | 0.007 | 8E-04 |
|  | Cohort 2 - MI | 0.692 | 0.845 | 0.586 | - | 0.002 |
|  | Cohort 2 - NY | 0.748 | 0.863 | 0.649 | 0.705 | - |
